# Auto-Exposure Algorithm for Enhanced Mobile Robot Localization in Challenging Light Conditions

**DOI:** 10.3390/s22030835

**Published:** 2022-01-22

**Authors:** Marc-André Bégin, Ian Hunter

**Affiliations:** Massachusetts Institute of Technology, Department of Mechanical Engineering, Cambridge, MA 02139, USA; ihunter@mit.edu

**Keywords:** auto-exposure, visual odometry, simultaneous localization and mapping, robot vision

## Abstract

The success of robot localization based on visual odometry (VO) largely depends on the quality of the acquired images. In challenging light conditions, specialized auto-exposure (AE) algorithms that purposely select camera exposure time and gain to maximize the image information can therefore greatly improve localization performance. In this work, an AE algorithm is introduced which, unlike existing algorithms, fully leverages the camera’s photometric response function to accurately predict the optimal exposure of future frames. It also features feedback that compensates for prediction inaccuracies due to image saturation and explicitly balances motion blur and image noise effects. For validation, stereo cameras mounted on a custom-built motion table allow different AE algorithms to be benchmarked on the same repeated reference trajectory using the stereo implementation of ORB-SLAM3. Experimental evidence shows that (1) the gradient information metric appropriately serves as a proxy of indirect/feature-based VO performance; (2) the proposed prediction model based on simulated exposure changes is more accurate than using γ transformations; and (3) the overall accuracy of the estimated trajectory achieved using the proposed algorithm equals or surpasses classic exposure control approaches. The source code of the algorithm and all datasets used in this work are shared openly with the robotics community.

## 1. Introduction

The success of robot localization methods based on vision relies on the quality of the camera exposure. While many methods exist to mitigate poor exposure effects after images have been acquired (e.g., motion blur [1,2,3,4,5,6,7], saturation [8], low contrast [9,10,11]), these often jeopardize the real-time capabilities of state estimation. Moreover, the performance of these specialized visual odometry (VO) and simultaneous localization and mapping (SLAM) pipelines can only be less than or equal to some equivalent generic pipelines fed with appropriately acquired images. Under challenging light conditions, such as when dealing with HDR scenes, non-static illumination, or low-light conditions, the appropriate selection of an image exposure time and gain is therefore crucial. However, in comparison to the methods mentioned above, pre-acquisition methods such as auto-exposure (AE) algorithms have received relatively little attention in the robot vision community. This is due in part to the fact that fine-tuning a specialized VO/SLAM pipeline can be achieved using a small set of prerecorded videos. In comparison, tuning an auto-exposure algorithm for maximizing VO performance requires more elaborate testing procedures such as replicating a camera trajectory multiple times under different parameter settings. As summarized in Table 1, existing AE algorithms mainly differ in the following aspects: (1) the metric optimized by the algorithm; (2) the model used to predict the effect of future changes in gain and exposure; (3) the strategy employed to balance gain and exposure time; (4) the control policy used to update the exposure parameters. As the merits and shortcomings of existing methods can be attributed to each of these individual aspects, they are addressed sequentially in the following sections.

### 1.1. Optimization Metrics

In the context of vision-based robot localization, the metric optimized by an AE algorithm acts as a proxy to the overall VO performance. The value of these optimization metrics depends on the exposure of the image. In this work, the exposure is quantified using the exposure level *E*, defined as

(1) $$E=\log_{2}\left(tg\right)=\log_{2}t+\left(\frac{\log_{2}10}{20}\right)g_{\mathrm{dB}},$$

where *t* and *g* are the exposure time and gain, and $g_{\mathrm{dB}}$ is the gain in dB. One common optimization metric is the deviation between some reference value and the average pixel intensity over the whole image or some region of interest (ROI) [12,14,21]. Minimizing this deviation is the most common AE approach available on most commercial off-the-shelf cameras. Although the metric is fast to compute, it is more useful for acquiring visually appealing images than for VO applications. Instead, AE algorithms for robot vision typically maximize the content of the image which is specifically relevant for feature detection and tracking. For instance, early work by Pan [22] in the field of autonomous driving maximizes the mean difference in intensity between lane markings and the road. A more general algorithm was later proposed by Lu [13] which maximizes image entropy, the assumption being that images with high entropy are well exposed. The image entropy metric $M_{e}$ is defined in this context as

(2) $$M_{e}=-\sum_{i=1}^{N_{l}}P_{i}\log_{2}P_{i},$$

where $P_{i}$ is the proportion of pixels in the image with an intensity value *i* out of the $N_{l}$ possible levels. Shim [16,23] later observed that images with strong gradients are more likely result in features being detected and matched. To balance the relative weight of weak and strong image gradients and to limit sensitivity to noise, Shim proposed that the gradient information $m_{i}^{*}$ of a pixel *i* should be defined as

(3) $$m_{i}^{*}=\left\{\begin{array}{cc} \frac{1}{N}\log\left(\lambda (m_{i}-\delta )+1\right), & \mathrm{if}\;m_{i}\geq \delta ,\\ 0 & \mathrm{otherwise}, \end{array}\right.$$

where $m_{i}$ is the gradient magnitude of the $i^{\mathrm{th}}$ pixel (normalized on a unit scale), $\delta \in R^{+}$ is the activation threshold, $\lambda \in R^{+}$ is a shaping parameter, and *N* is a normalization factor defined as

(4) N=log(λ(1−δ)+1).

**Figure 1:**
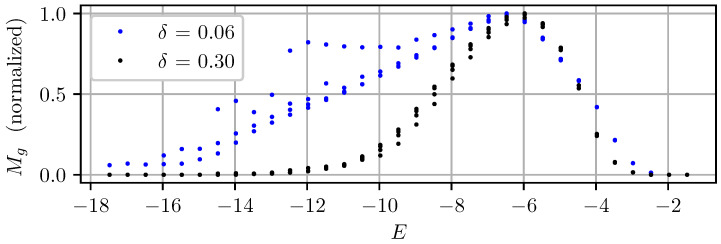
Comparison of the gradient information metric $M_{g}$ for different threshold values δ. Each dot represents the metric computed for an image of a static scene with a constant illuminance of about 50 lux captured with different gains and exposure times.

Finally, the gradient information metric $M_{g}$ is defined as

(5) $$M_{g}=\sum_{i=1}^{N_{p}}m_{i}^{*},$$

where $N_{p}$ is the total number of pixels in the image. As proposed in Shim’s original paper, λ was set in this work to 1000 [16]. The activation threshold δ was set to 0.30, which is larger than the value of 0.06 originally used by Shim. Indeed, a desired feature of the metric is that it should be independent of image noise. As image noise increases with gain, the metric value should not vary when acquiring the image of a static scene at a given exposure level *E*, even if the image is acquired with different combinations of exposure gain and time. When using an activation threshold of 0.06, experimentation showed that the image noise in dark frames causes $M_{g}$ to vary greatly for different gains (see Figure 1). In comparison, a threshold of 0.30 decouples metric values from image noise while the optimal exposure level remains the same.

Zhang [15] later introduced a closely related metric labeled the “soft” gradient percentile. It approximates a certain percentile of the image pixel gradients (e.g., the median gradient) and, unlike gradient information, it is differentiable with respect to exposure time. This property proves useful in deriving a controller policy based on gradient descent.

However, $M_{e}$, $M_{g}$, and the median gradient can be sensitive to noise. To avoid the problem, Kim [17] proposed to weight the gradient of each pixel *i* by a factor which depends on the entropy $e_{i}$ of the pixel defined as

(6) $$e_{i}=-P\left(I_{i}\right)\log_{2}P(I_{i}),$$

where $P(I_{i})$ is the proportion of pixels in the image with the same intensity as pixel *i*. The weight $W_{i}$ of a pixel is then defined as

(7) $$W_{i}=\frac{w_{i}}{\sum_{j=1}^{N}w_{j}},$$

where

(8) $$w_{i}=\frac{1}{\sigma_{e}}\exp\left(-\frac{1}{2}{\left(\frac{e_{i}-\bar{e}}{\sigma_{e}}\right)}^{2}\right),$$

and where $\bar{e}$ and $\sigma_{e}$ are, respectively, the mean pixel entropy and standard deviation. Finally, the authors define the entropy-weighted gradient ${\overset{ˇ}{m}}_{i}$ of a pixel as

(9) $${\overset{ˇ}{m}}_{i}=W_{i}m_{i}^{2}+\pi \left(e_{i}\right)S_{i}W_{i}\frac{1}{N_{p}}\sum_{j=1}^{N_{p}}m_{j}^{2},$$

where

(10) $$S_{i}=\left\{\begin{array}{cc} 1 & \mathrm{if}\;e_{i}\leq e_{\mathrm{thresh}},\\ 0 & \mathrm{otherwise}, \end{array}\right.$$

and π(·) is an activation function defined as

(11) $$\pi \left(e_{i}\right)=\frac{2}{1+\exp\left(-\alpha e_{i}+\tau \right)}-1,$$

where $\alpha \in R^{+}$ and $\tau \in R^{+}$ are shaping factors. For the present work, $e_{\mathrm{thresh}}=0.05$, α=32 and τ=4 as in [17]. The entropy-weighted gradient metric $M_{ewg}$ is then computed as

(12) $$M_{ewg}=\sum_{i=1}^{N_{p}}{\overset{ˇ}{m}}_{i}.$$

As an alternative to Kim’s weighted gradient, Shin [18] proposed that the noise $\sigma_{\mathrm{noise}}$ of an image (I) be directly approximated as

(13) $$\sigma_{\mathrm{noise}}=\sqrt{\frac{\pi }{2}}\frac{1}{N_{s}}\sum_{i=1}^{N_{p}}H_{i}\cdot U_{i}\cdot {\left|I*M\right|}_{i},$$

where $H_{i}$ and $U_{i}$ are the $i^{\mathrm{th}}$ entry of binary matrices, respectively, masking out the non-homogeneous and the saturated regions of the image, $N_{s}$ is the number of pixels that are both homogeneous and unsaturated, and M is the noise estimation kernel proposed by Immerkaer [24]. The approximation $\sigma_{\mathrm{noise}}$ of the image noise is then incorporated in a hybrid image quality metric $M_{q}$ defined as

(14) $$M_{q}=\alpha \frac{M_{g}}{s_{g}}+\left(1-\alpha \right)M_{e}-\beta \sigma_{\mathrm{noise}},$$

where $\alpha \in \left(0,1\right)$ and $\beta \in R^{+}$ are weighting factors, and $s_{g}$ is the standard deviation of the gradient information metric evaluated individually over each cell of a grid. The present work uses the authors’ original values of α=0.4 and β=0.4 proposed in [18].

More recently, Tomasi [20] also proposed to directly maximize the number of detected and successfully matched features across frames as a proxy for VO performance through a self-supervised learning method. However, from the current state of the literature, it remains unclear how these metrics compare to one another as substitutes of VO performance, since direct cross-correlation analyses are rarely offered. Furthermore, other metrics, such as the Lowe ratio has not yet been incorporated into an AE algorithm. Its merits as a proxy of VO performance are worth exploring in this work.

### 1.2. Prediction Models

While purely reactive approaches (e.g., [12,13,18]) do not require any characterization of the camera, they do require the slow process of sampling real-world images to converge. Instead, predictive AE algorithms use a model to predict the effect of future exposure parameters on the optimization metric. For learned control policies (e.g., [14,17,20]), a predictive model is implicitly embedded in the policy. In contrast, explicit predictive methods offer more interpretability, and leverage known information about the camera’s image acquisition process. For instance, Shim [16,23] proposed to use discrete γ transformations to predict the effects of future variations of exposure parameters. For each pixel intensity $I_{\mathrm{in}}\in \left\{0,1,\ldots ,255\right\}$ of an image and for any given $\gamma \in R^{+}$, the predicted pixel intensity $I_{\mathrm{out}}\in \left\{0,1,\ldots ,255\right\}$ is mapped as

(15) $$I_{\mathrm{out}}=255\cdot {\left(\frac{I_{\mathrm{in}}}{255}\right)}^{\gamma }.$$

This approach does not require any camera characterization, but there exists no direct link between γ transformations and changes in camera exposure other than γ<1.0 simulates a more exposed image while γ>1.0 simulates a less exposed one. To avoid this limitation, Zhang [15] proposed to leverage the photometric response function (PRF) of the camera as a way to make better predictions. This function $f_{\mathrm{PRF}}$ maps the exposure *E* of the camera sensor to the intensity $I_{\mathrm{out}}$ of the image:

(16) $$I_{\mathrm{out}}=f_{\mathrm{PRF}}\left(E\right).$$

The PRF of a camera can be found through a simple calibration procedure [25] or be estimated online [26], but only up to an offset which depends on the unknown scene irradiance. While Zhang leverages this PRF within a gradient descent step to select the next exposure parameters, it has not been incorporated into an explicit prediction step similar to Shim’s algorithm.

### 1.3. Gain/Exposure Balance Strategies

From the definition of the exposure level *E* given in (1), there are infinitely many combinations of exposure time and gain that will result in the same exposure level. Most existing AE algorithms (e.g., [12,15,16,19]) disambiguate this choice using an “exposure priority”. With this approach, the exposure time is always adjusted first while maintaining gain at a fixed low value. When exposure time reaches an upper limit, gain is increased to meet the required exposure level, thus minimizing image noise. In low-light conditions, this method is seriously prone to motion blur. One way to solve the issue is to impose some fixed relationship between *g* and *t* (e.g., g=kt, where $k\in R^{+}$) [13]. However, such a relationship is suboptimal as it does not allow motion blur and noise to be balanced dynamically based on the current motion of the camera.

### 1.4. Control Policies

Almost every algorithm summarized in Table 1 employs a different control policy. Yet, the merits of each (apart from allowing some predictive control or leveraging some learning method) cannot realistically be compared in isolation from the choice of optimization metric, prediction model, and gain/exposure balance strategy. Among these, Shim’s AE algorithm [16,19] stands apart by its use of an explicit prediction model which enables the quick convergence of the camera exposure parameters with a simple feedback law. It is also the method closest to the one proposed in this work.

For every incoming frame, the authors simulate changes in exposure by applying a sequence of discrete γ transformations (15) to the input image. The gradient information metric of each simulated image is then computed and a polynomial function $f_{\mathrm{fit}}\left(\gamma \right)$ is fit to the resulting data. The optimal transformation is then approximated as $\gamma^{*}=\arg\max f_{\mathrm{fit}}\left(\gamma \right)$. As there exists no direct relationship between γ transformations and changes in exposure level, the authors then set the exposure level of the next frame $E_{k+1}$ using the nonlinear update

(17) $$E_{k+1}=\left(1+\alpha k_{p}\left(r-1\right)\right)E_{k},$$

where

(18) $$r=d\cdot \tan\left(\arctan\left(1/d\right)\left(1-\gamma^{*}\right)\right)+1,$$

and

(19) $$\alpha =\left\{\begin{array}{cc} 1/2 & \mathrm{if}\;\gamma^{*}\geq 1,\\ 1 & \mathrm{otherwise}. \end{array}\right.$$

### 1.5. Proposed Approach

The aim of the present work is to detail and support the development of an auto-exposure algorithm for the purpose of vision-based robot localization in challenging light conditions. Unlike other methods, the algorithm detailed in Section 2.1 fully leverages the camera’s PRF to predict the exposure that maximizes gradient information. It also incorporates feedback on the error between the actual and predicted gradient information metrics to compensate for PRF inaccuracies due to image saturation. Finally, it balances gain and exposure time based on time-varying predictions of motion blur intensity. Using the setup and testing procedure detailed in Section 2.2, the overall performance of the algorithm is assessed through extensive experimental validation. First, a cross-correlation analysis (Section 3.1) for a wide range of optimization metrics supports the use of gradient information as an appropriate proxy of VO performance. A convergence analysis (Section 3.2) then demonstrates the respective effects of using predictions based on PRF and feedback to compensate prediction errors due to saturation. Finally, the AE algorithm’s ability to reduce robot localization error is assessed experimentally in Section 3.3 and demonstrates that the proposed approach outperforms other exposure control approaches.

## 2. Materials and Methods

### 2.1. Proposed AE Algorithm

**Figure 2:**
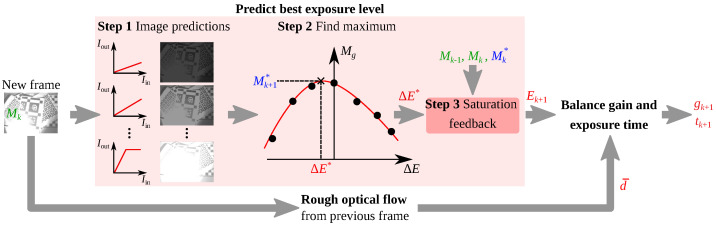
Schematic of the AE algorithm proposed in the present work.

The proposed algorithm actively adjusts the camera gain and exposure time to improve VO performance by maximizing the image gradient information metric (5). While the algorithm can handle any proxy of VO performance, this choice of metric is supported by the detailed comparison included in Section 3.1. A schematic and pseudocode summarizing the algorithm are provided in Figure 2 and Algorithm 1. The C++ source code and a ROS wrapper for this algorithm are made publicly available (https://github.com/MIT-Bilab/vo-autoexpose accessed on 30 November 2021). The code also includes options to experiment with the different alterations of the algorithm tested in this work. It supports, for instance, Shim’s prediction model based on γ transformations by reusing some portions of the open-source code shared by Mehta [19].
**Algorithm 1** AE for challenging light conditions.  1:**function**RoughOpticalFlow($I_{k-1}$, $I_{k}$)  2: ${\overset{ˇ}{I}}_{k-1},{\overset{ˇ}{I}}_{k}\leftarrow$ Downsize $I_{k-1}$ and $I_{k}$ (e.g., 90 × 68)  3: Δx,Δy← Compute Farneback optical flow from $I_{k}$ and $I_{k-1}$  4: $\bar{d}\leftarrow \frac{1}{n_{p}}\sum_{i=1}^{n_{p}}\sqrt{\Delta x_{i}^{2}+\Delta y_{i}^{2}}$  5: **return** $\bar{d}$  6:**end function**  7:   8:**function**PredictBestExposureLevel($I_{k},t_{k},g_{k},M_{k}^{*}$)  9: **for** $i\leftarrow 1,n_{\mathrm{predictions}}$ **do**                              ▷ **Step 1**: Image predictions10:  $I_{\mathrm{predict}}\leftarrow$ Predict image based on $\Delta E_{i}$ using lookup table *i*11:  $p_{i}\leftarrow$ Compute gradient information from $I_{\mathrm{predict}}$ using Sobel operators and (5)12: **end for**13: 14: $f_{\mathrm{fit}}\left(\Delta E\right)\leftarrow$ Compute 6th degree polynomial least-square approximation of p=f(ΔE)  ▷ **Step 2**: Find maximum15: $\Delta E^{*}\leftarrow 0$16: **while** $\left|\delta \right|>$ threshold **do**17:  $\delta \leftarrow f_{\mathrm{fit}}^{\prime }\left(\Delta E^{*}\right)\;/\;f_{\mathrm{fit}}^{\prime \prime }\left(\Delta E^{*}\right)$18:  $\Delta E^{*}\leftarrow E^{*}+\delta$19: **end while**20: 21: $M_{k}\leftarrow$ Compute gradient information of $I_{k}$ according to (5)              ▷ **Step 3**: Saturation feedback22: $M_{k+1}^{*}\leftarrow$ Predict gradient information of next frame as $f_{\mathrm{fit}}\left(\Delta E^{*}\right)$23: $E_{k+1}\leftarrow$ Compute according to (22) using $M_{k-1}$, $M_{k}$, $M_{k}^{*}$, and $\Delta E^{*}$24: 25: **return** $E_{k+1}$, $M_{k+1}^{*}$26:**end function**27: 28:**function**BalanceGainExposureTime($E_{k+1}$, $\bar{d}$)29: $g_{k+1}\leftarrow$ Compute according to (25)30: $t_{k+1}\leftarrow$ Compute according to (26)31: $t_{k+1}$, $g_{k+1}\leftarrow$ If $t_{k+1}$ is out of bounds, compensate with $g_{k+1}$ (and vice-versa)32: **return** $t_{k+1}$, $g_{k+1}$33:**end function**34: 35:k←1                                         ▷ **Main algorithm loop**36:**loop**37: $I_{k},t_{k},g_{k}\leftarrow$ Pull new camera frame38: $I_{k}\leftarrow$ Downsize $I_{k}$ (e.g., 360 × 270)39: $I_{k}\leftarrow$ Apply median blur to $I_{k}$40: $E_{k+1},M_{k+1}^{*}\leftarrow$PredictBestExposureLevel($I_{k},t_{k},g_{k},M_{k}^{*}$)41: **if** k > 1 **then**42:  $\bar{d}\leftarrow$RoughOpticalFlow($I_{k-1},I_{k}$)43: **end if**44: $t_{k+1},g_{k+1}\leftarrow$BalanceGainExposureTime($E_{k+1},\bar{d}$)45: k←k+146:**end loop**

**Figure 3:**
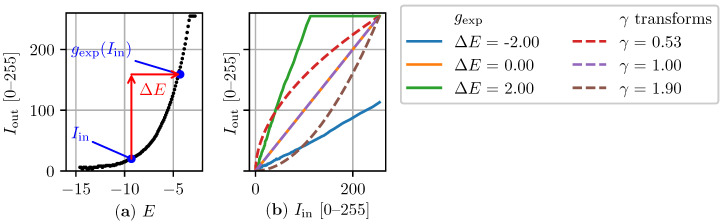
Example of a camera’s photometric response function and different corresponding $g_{\exp}$ transformations: (**a**) The photometric response function of the camera relates the intensity of a pixel to its exposure level. For any given change in exposure ΔE, a pixel intensity $I_{\mathrm{in}}$ always has a corresponding transformed pixel $g_{\exp}\left(I_{\mathrm{in}}\right)$. (**b**) The $g_{\exp}$ transformations relate the pixel intensity of an input image $I_{\mathrm{in}}$ to its predicted intensity $I_{\mathrm{out}}$ given a certain change in exposure level ΔE. The mapping of these transformations is obtained directly from the camera’s photometric response function. Different γ transformations (15) are also shown for comparison.

For every new camera frame $I_{k}$, the main algorithm loop consists of first predicting the best exposure level $E_{k+1}$ of the next frame. This process can be broken down into three main steps. In **Step 1**, $n_{\mathrm{predictions}}$, discrete changes in exposure level are artificially applied to the image. Unlike Shim’s method, which uses predictions based on γ transformations, changes in exposure level can only be predicted if the camera’s PRF is available. Figure 3a, for instance, shows the PRF of the camera used in this work. It relates the exposure level of any pixel to its intensity and can be obtained from a simple static calibration procedure [25]. The function is only defined up to some offset in *E* which depends on the illumination of the scene. Given the camera’s PRF and for any given change in exposure level ΔE, one can approximate a monotonically increasing function $g_{\exp}$ similar to the γ transformation (15) which maps the intensity value $I_{\mathrm{in}}$ of every pixel in the input image to its predicted value $I_{\mathrm{out}}$:

(20) $$I_{\mathrm{out}}=g_{\exp}\left(I_{\mathrm{in}}\right).$$

Examples of $g_{\exp}$ transformations for different changes in exposure level are provided in Figure 3b. The figure also includes examples of γ transformations to illustrate the difference. A justification for using $g_{\exp}$ instead of γ transformations relies on the detailed comparison included in Section 3.2. **Step 1** terminates with the calculation of the gradient information metric of each simulated image $I_{\mathrm{predict}}$. The metric of the $i^{\mathrm{th}}$ simulated image is denoted as $p_{i}$.

**Step 2** consists of estimating the change in exposure level $\Delta E^{*}$ that maximizes gradient information. A least-squares 6th degree polynomial approximation $f_{\mathrm{fit}}$ is fit through the metrics of the simulated images. Newton’s iterative method initialized at the origin is then used to approximate $\Delta E^{*}$ as the maximum argument of $f_{\mathrm{fit}}$. This step is largely inspired by Shim’s approach to find the optimal $\gamma^{*}$ transformation, as described in Section 1.4.

**Figure 4:**
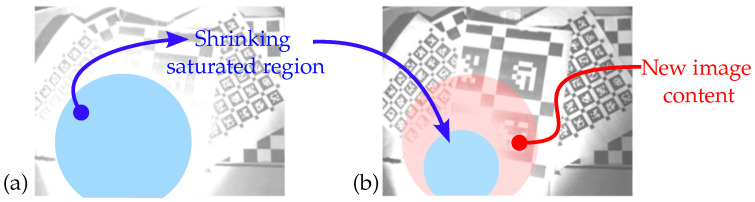
Example of a static scene for which changes in pixel intensity cannot be accurately predicted by $g_{\exp}$. (**a**) Scene with a single large overexposed region. (**b**) The same scene with a smaller overexposed region revealing image content not predicted by $g_{\exp}$.

Finally, **Step 3** aims to compensate prediction errors due to image saturation. Indeed, saturated pixels cannot accurately be mapped by $g_{\exp}$, which is problematic for large and sudden changes in lighting conditions (e.g., lights turning on/off). Under such circumstances, the $g_{\exp}$ transformation systematically underestimates changes in exposure level which could greatly improve gradient information by unsaturating some part of an image. This leads to smaller steps $\left|\Delta E\right|$ and a longer convergence time. To circumvent some of the issue, the proposed strategy is to artificially increase $\left|\Delta E\right|$ when the improvement in the gradient information metric from one frame to the next is substantially better than predicted (e.g., Figure 4).

Let *r* be the ratio of the actual change in the gradient information metric over the predicted one:

(21) $$r=\frac{M_{k}-M_{k-1}}{M_{k}^{*}-M_{k-1}+\epsilon },$$

where $\epsilon \in R^{+}$ is a relatively small number (e.g., ϵ=0.1) and $M_{k}^{*}=f_{\mathrm{fit}}\left(\Delta E^{*}\right)$. Then, the exposure level $E_{k+1}$ of the next frame is selected as

(22) $$E_{k+1}=\left\{\begin{array}{cc} E_{k}+\Delta E^{*} & \mathrm{if}\;r<r_{\mathrm{th}},\\ E_{k}+f_{d}\Delta E^{*} & \mathrm{otherwise}, \end{array}\right.$$

where $f_{d}>1.0$ is a constant factor on the step size and $r_{\mathrm{th}}\in R^{+}$ is the threshold of *r* (e.g., $f_{d}=1.5$, $r_{\mathrm{th}}=1.1$ were used in this work).

Once the desired exposure level for the next frame $E_{k+1}$ is set, explicit values for the camera gain $g_{k+1}$ and exposure time $t_{k+1}$ still need to be selected. It is well known that image noise increases with gain, that motion blur increases with exposure time, and that both negatively affect localization. The relative importance of each effect largely depends on the specific VO/SLAM algorithm used. Feature-based algorithms, such as ORB-SLAM [27] and VINS-Fusion [28], for instance, are more strongly affected by motion blur and less affected by noise than methods such as DSO [29], which minimizes photometric error. The proposed algorithm exploits a simple way to balance gain $g_{k+1}$ and exposure time $t_{k+1}$ based on a single constant factor $w\in R^{+}$, weighting the relative importance of image noise and motion blur as

(23) $$\begin{array}{cc} \underset{t_{k+1},g_{k+1}}{\arg\min}\;\;\; & \left({\bar{d}}^{2}+\frac{k_{\mathrm{offset}}}{c}\right)t_{k+1}+w\left(g_{k+1}-1\right)\\ s.t.\;\;\; & t_{k+1}\cdot g_{k+1}=c, \end{array}$$

where $k_{\mathrm{offset}}\in R^{+}$ is a constant scalar ($k_{\mathrm{offset}}=8$ in this work), $\bar{d}$ is the average speed of image points (pixels/second), which can be approximated with Farneback’s optical flow method [30], and, from (1),

(24) $$c=2^{E_{k+1}}.$$

Hence, the procedure associates a cost that grows quadratically with the average motion blur length and linearly with gain. This choice of the exponents for the cost function is consistent with the experimental characterization of ORB-SLAM3 included in Section 3.1. It shows that ORB-SLAM3 is more sensitive to motion blur than image noise (gain) and that the rate at which it degrades increases with exposure time. Hence, the cost function (23) is specific to ORB-SLAM3 and might not be appropriate for direct VO methods such as DSO [29]. One should recharacterize the sensitivity of the method with respect to noise and motion blur before deciding on specific exponents. The choice of hyperparameter *w* also depends on the selected VO algorithm. In this work, *w* was hand-tuned for ORB-SLAM3. Starting with a unit value, *w* was gradually decreased over multiple test sequences until a peak performance was reached around w=0.02. Indeed, the AE algorithm can become unstable for small values of *w* as the exposure parameters vary too quickly. When using the AE algorithm with other VO methods, the same procedure should be repeated to tune *w*. For instance, DSO [29] is a direct VO method which is more sensitive to image noise than feature-based methods like ORB-SLAM3.

If the minimization problem (23) is feasible, then it admits the unique solution

(25) $$g_{k+1}=\sqrt{\frac{{c\bar{d}}^{2}+k_{\mathrm{offset}}}{w}},\;\mathrm{and}$$

(26) $$t_{k+1}=\frac{c}{g_{k+1}}.$$

This solution depends on the average speed of image points $\bar{d}$ determined by optical flow. For small values of $\bar{d}$, the method selects images with a low gain, thus minimizing noise. For large values of $\bar{d}$, the method selects images with a low exposure time, thus minimizing motion blur. Unlike existing AE methods, the one proposed thus leverages optical flow to select exposure parameters that are optimal given the current motion of points in the image.

### 2.2. Experimental Setup

**Figure 5:**
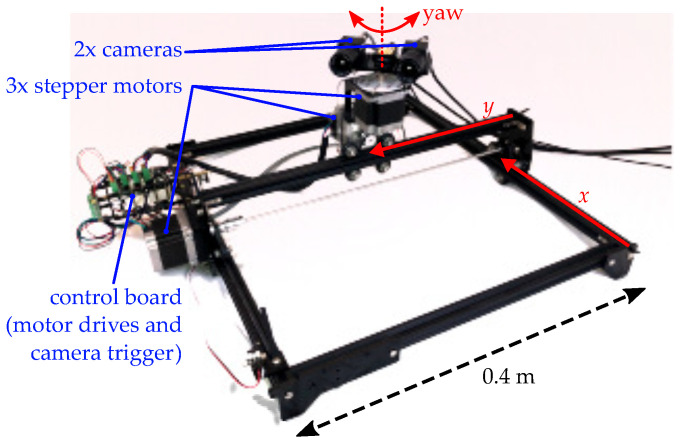
Picture of the custom experimental setup consisting of a stereo camera setup mounted on a three-axis (xy–yaw) motion table.

The experimental setup used in this work and shown in Figure 5 comprises two monochrome machine vision cameras (FLIR BFS-U3-16S2M) 80 mm apart and simultaneously triggered at 60 Hz. Images are acquired at a 720 × 540 resolution using a 2 × 2 binning in order to increase light sensitivity. All experiments are performed with the cameras mounted on a custom three-axis (xy–yaw) motion table. Each axis is actuated by a NEMA 23 stepper motor. The drives used to power each motor (Tinkerforge silent stepper bricks) also provide a ground truth trajectory with a precision of ~0.1 mm. The cameras communicate over USB3 to a separate computer which runs the proposed AE algorithm in real time at 60 Hz on a single core of an AMD Ryzen 7 3700x CPU.

**Figure 6:**
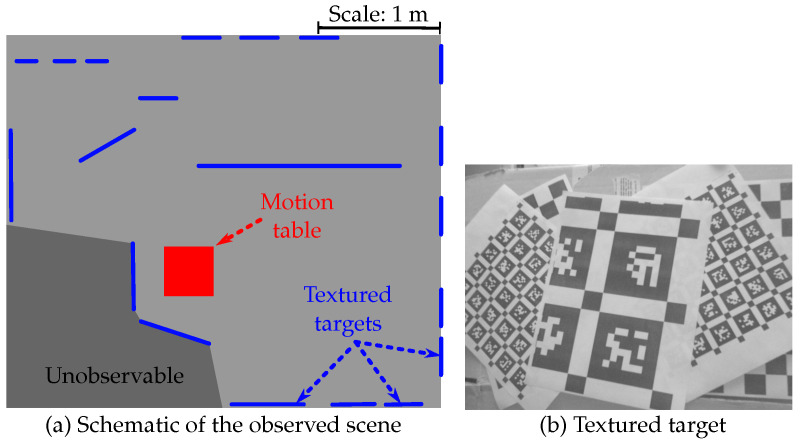
Details of the scene observed during the tests: (**a**) Schematic of the observed scene showing the approximate placement of the textured targets placed around the room. (**b**) Sample image of a textured target placed in the room.

A top-view schematic of the static scene observed throughout the experiments is shown in Figure 6a. Targets with a unique texture (e.g., AprilTags), such as shown in Figure 6b, were plastered throughout the room in order to prevent tracking failures of the VO algorithm. The distance between the camera and these targets varies during the recording between approximately 0.2 m and 2.5 m.

## 3. Results

### 3.1. Benchmarking Proxies of VO Performance

**Figure 7:**
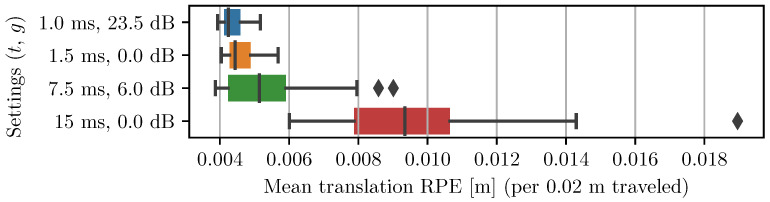
VO performance acquired with the three-axis motion table for different fixed exposure parameters. Each pair of parameters (exposure time, gain) is used over four recordings. Each recording is post-processed 10 times with ORB-SLAM. Failed runs (tracking lost) are not included.

**Figure 8:**
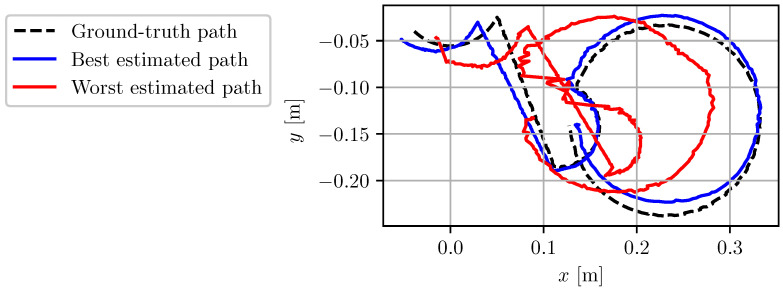
Top view of the ground-truth path followed by the left camera throughout all of the test sequences. The best and worst paths estimated by ORB-SLAM3 are overlaid for comparison.

In order to benchmark the different proxies of VO performance introduced in Section 1.1, the motion table was commanded to execute a preset path. The maximum linear speed reached over the trajectory is 100 mm/s and the maximum rotation speed is 100 deg/s. Each frame of the left camera video feed was preprocessed with a median blur filter and a static γ transformation of 0.3 to enhance image contrast. Four different static exposure settings were used. For each setting, the trajectory was repeated four times. The video feed of the cameras for each repetition was then post-processed 10 times with ORB-SLAM3 [27] in stereo mode without loop closures. Each estimated trajectory was then compared against the ground-truth trajectory, and VO performance was measured using the mean translation relative pose error (RPE) computed over 20 mm sub-trajectories [31]. The VO performance for the four static camera exposure settings is presented in Figure 7. It shows that increasing the exposure time both increases the median RPE and the spread of the results. It also shows that ORB-SLAM is relatively insensitive to image noise due to high gain. This supports choosing an AE algorithm with a gain/exposure balance strategy that favors low exposure times (small *w*). The best and worst trajectories estimated by the VO algorithm are also overlaid over the ground-truth trajectory of the left camera in Figure 8.

**Figure 9:**
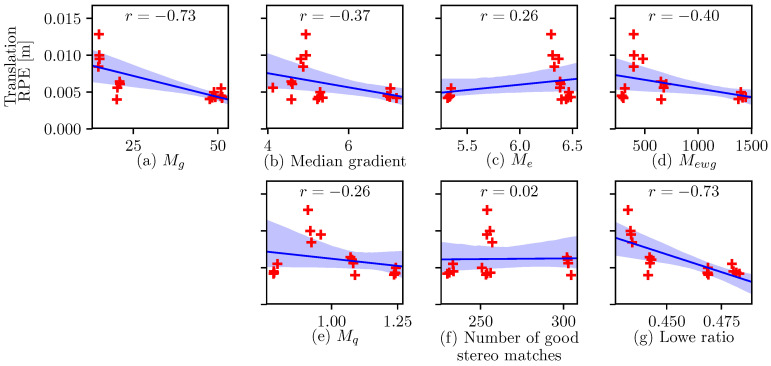
Linear regression and 95% confidence region for different candidate proxies of VO performance: (**a**) Gradient information metric (5). (**b**) Median gradient. (**c**) Entropy metric (2). (**d**) Entropy-weighted gradient metric (12). (**e**) Quality metric (14). (**f**) Number of good stereo matches. (**g**) Lowe ratio for the stereo matches.

Then, for each image of the video sequence recorded by the left camera, the following metrics were computed: the gradient information metric $M_{g}$, the gradient median (similar to Zhang’s “soft” percentile metric [15]), the entropy metric $M_{e}$, the entropy-weighted gradient metric $M_{ewg}$, and the quality metric $M_{q}$. In addition, from the synchronized left and right camera frames, the number of good stereo matches and the Lowe ratio for these matches were also computed. For each sequence, the median of the metric over the whole sequence is plotted in Figure 9 against the corresponding average translation RPE. The gradient information metric (a) and Lowe ratio (g) show the best correlations with VO error (r=−0.73 for both). As computing the Lowe ratio is more computationally expensive, Shim’s gradient information was selected as the optimization metric for the AE algorithm proposed in this work.

Although the median gradient (Figure 9b) is closely related to the gradient information metric, image noise can artificially increase its value, which explains the lower correlation (r=−0.37). In comparison, the thresholding function (3) used in the definition of the gradient information metric allows to mitigate this bias, as detailed in Section 1.

In [13], the authors show that image entropy is maximized when the number of under- or overexposed pixels of an image is minimized. The authors then show that under static conditions, images selected based on entropy lead to better localization compared to some static exposure parameters. However, out of all the tested metrics in the present work, image entropy (Figure 9c) has the worst cross-correlation with VO error (r=0.26). This indicates that while the metric might limit the number of saturated pixels, it fails to properly capture the detrimental effects of motion blur and noise. Another explanation for this poor result is that, in some cases, it might also be beneficial to allow some parts of the image to be under- or overexposed in order to highlight more informative regions of the image.

The entropy-weighted gradient $M_{\mathrm{ewg}}$ (Figure 9d) was initially proposed in [17] to minimize noise effects on the image gradient. As such, the cross-correlation achieved by the metric (r=−0.40) is slightly better than that achieved with the median gradient. Yet, it is still far from the cross-correlation achieved with the gradient information metric. This supports that a thresholding function such as (3) is more effective for removing noise effects than weighting the image gradient with entropy.

Another way to limit sensitivity to noise was proposed in [18]. As detailed in Section 1.1, the authors introduced the quality metric $M_{q}$ (Figure 9e). This metric is a hybrid metric between gradient information and entropy from which a weighted estimation of the image noise intensity is subtracted. Here, again, the poor cross-correlation achieved by the metric (r=−0.26) indicates that a thresholding function such as (3) is more effective for removing noise effects than subtracting the estimated noise intensity from the image metric. This low cross-correlation is also, in part, explained due to $M_{q}$ directly incorporating the entropy metric $M_{e}$, which does not correlate to VO error.

For the test sequences used in the present work, the cross-correlation between the number of good stereo matches (Figure 9f) and VO error was almost nonexistent (r=0.02). These good stereo matches were determined by first detecting ORB features in each corresponding left and right images. Each feature in the left image was then matched to one in the right image using the k-nearest neighbors algorithm. Matches were labeled as “good” if the stereo equipolar error was smaller than 1 pixel and if the Lowe ratio of the match was smaller than 0.7. Despite this outlier rejection scheme, image noise was still found to have a large impact on the number of good matches. However, feature tracking performance largely depends on the saliency of the features. Hence, unlike the raw number of good stereo matches, the median Lowe ratio of those good matches (Figure 9g) strongly correlates with VO error (r=−0.73).

### 3.2. Convergence Analysis

**Figure 10:**
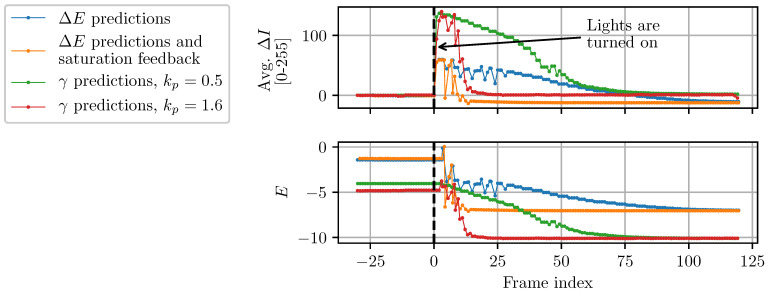
Step response of the proposed AE algorithm subject to an instantaneous increase in ambient light (from ~1 to ~150 lux). Frames are acquired and processed in real time at 60 Hz.

The AE algorithm proposed in this work uses a prediction model based on the camera’s PRF. It also incorporates feedback to correct for some of the prediction inaccuracies due to image saturations. As shown by the camera’s response to a step in ambient light (Figure 10), these choices have a drastic effect on the camera’s response time. For instance, without saturation feedback, the proposed algorithm can take up to 100 frames to converge compared to about 15 frames with feedback. Similar convergence speeds can be achieved using Shim’s control method [16,19], which incorporates a prediction model based on γ transformations. However, the two methods do not converge to the same exposure level.

**Figure 11:**
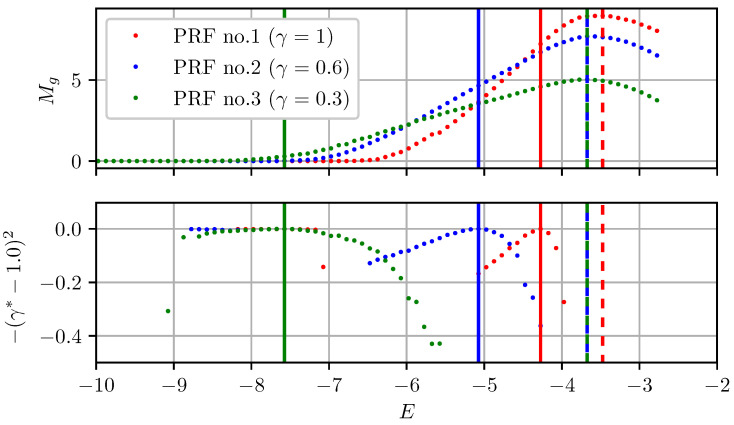
Gradient information metric for different exposure levels of the same static image. Each peak (marked with discontinuous vertical lines) corresponds to the true optimal exposure level. Peaks for the bottom graph (marked with continuous vertical lines) correspond to the optimal exposure levels predicted by Shim’s γ transformations [16]. Each colored curve represents a different camera’s PRF.

To investigate how γ transformations affect the predicted optimal exposure level, images of a static scene were acquired at different exposure levels (*E*). The gradient information metric ($M_{g}$) was then computed for each image as well as the optimal transformation $\gamma^{*}$ predicted by Shim’s method. Both are plotted as a function of exposure level in Figure 11. To simulate three different cameras’ PRF, the same procedure was repeated with static γ transformations applied to the incoming images with values of 0.6 and 0.3. The idea behind Shim’s approach is that for the exposure level corresponding to $\gamma^{*}=1$, $M_{g}$ should also be maximum. However, as can be seen from Figure 11, the procedure systematically underestimates the optimal exposure level. In comparison, the true optimal exposure level only slightly varies for different cameras’ PRF. A prediction model using PRF-based transformations therefore avoids this bias and selects exposure levels which are closer to the true optimal ones.

### 3.3. VO Performance

The performance of the proposed AE algorithm was tested and directly compared to using fixed exposure parameters, the camera’s built-in AE algorithm, and Mehta’s open-source implementation of Shim’s algorithm [19]. A supplementary video illustrating this comparison is available online (https://youtu.be/Guvhvb-uQpE accessed on 30 November 2021). The fixed exposure parameters were hand-tuned such that tracking would not be lost due to under- or over-saturation. The reference pixel intensity value tracked by the built-in AE algorithm was set to 20% of the maximum pixel intensity value. Using higher target values would result in some frames being overexposed, and tracking would be lost. To allow a fair comparison, Mehta’s original code was altered to use the gain/exposure balance strategy of this work. Settings for the nonlinear controller were also selected to obtain a convergence time similar to the method proposed in this work (i.e., $k_{p}=1.6$ and d=0.1, as demonstrated in Section 3.2).

For these tests, the maximum exposure time of the camera was set to 7.5 ms, which represents about half of the cameras’ sampling time at 60 Hz. All incoming images were again preprocessed with a median blur filter and a contrast-enhancing γ transformation of 0.3. Each exposure method was tested in two scenarios. For both scenarios, the camera underwent the same trajectory and the objects in the scene remained the same. However, in scenario (a), lighting varied greatly between the different regions of the image (1–217 lux), while in scenario (b), lighting remained relatively low and constant (2–4 lux).

**Figure 12:**
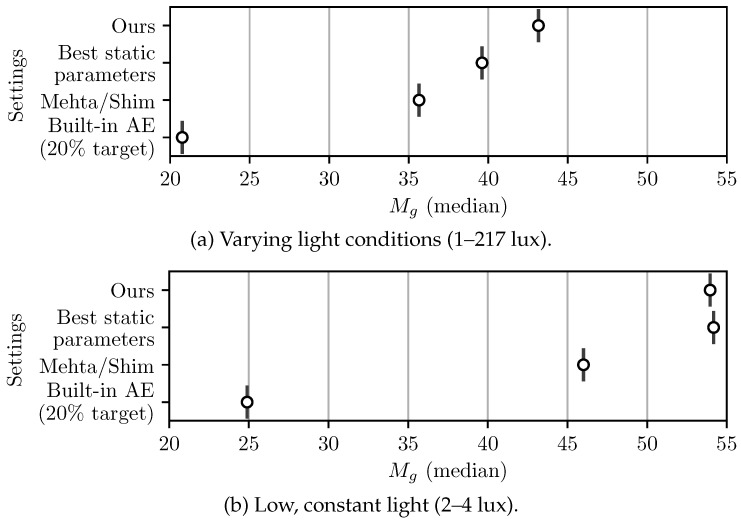
Median gradient information metric $M_{g}$ over the whole trajectory repeated using different AE algorithms: (**a**) Sequences where lighting varies greatly (1–217 lux). (**b**) Sequences with low, but constant lighting (2–4 lux).

**Figure 13:**
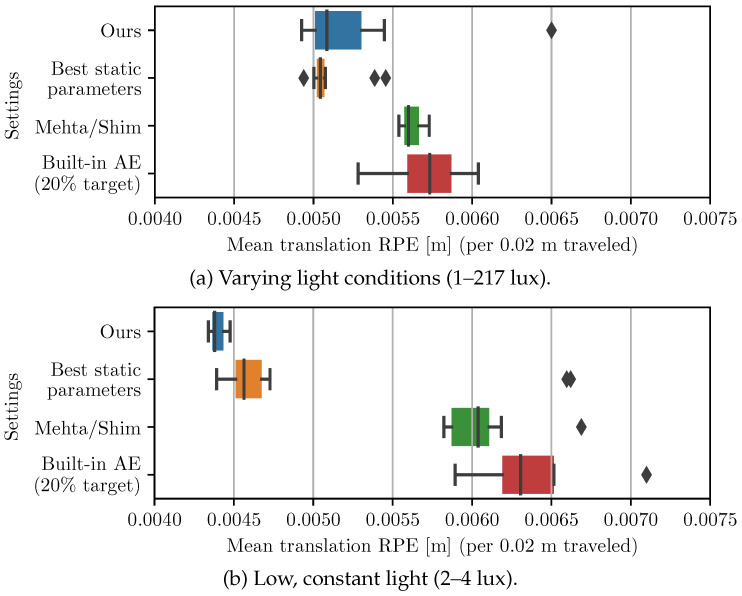
VO performance of different AE algorithms with the same repeated trajectory where each box represents the distribution of results for a sequence post-processed 10 times with ORB-SLAM: (**a**) Sequences where lighting varies greatly (1–217 lux). (**b**) Sequences with low, but constant lighting (2–4 lux).

As can be seen in Figure 12, the proposed AE algorithm systematically produces images with higher gradient metrics compared to the other active methods. The mean VO accuracy of each method are also compared in Figure 13 and demonstrate that the proposed algorithm achieves a lower tracking error. While the static parameters result in a similar performance to the proposed method in scenario (a), the same static parameters in (b) results in suboptimal performance. It should be mentioned that the test conditions (a) and (b) were chosen such that static parameters would generate images that a VO algorithm can track. Reusing the same parameters in drastically different light conditions (e.g., in sunlight) instead systematically results in VO failure.

**Figure 14:**
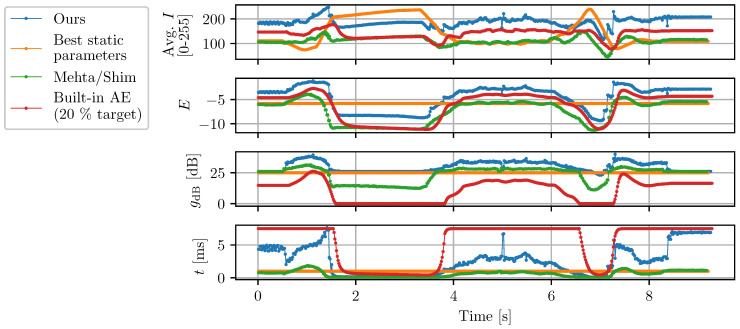
Comparison of the image exposure parameters selected by different AE algorithms. Light intensity captured by the cameras oscillates between 1 lux for the darker parts of the scene and 217 lux for the brighter parts (e.g., over the 1.5–3.5 s and 6.5–7.0 s intervals).

Finally, the exposure parameters selected by each exposure control method are compared in Figure 14 for scenario (a). As underlined in Section 3.2, Shim’s control method (which uses predictions based on γ transformations) underestimates the optimal camera exposure, leading to suboptimal VO performance.

## 4. Discussion

### 4.1. Optimization Metrics

One of the main differences between existing AE algorithms is the metric being (implicitly or explicitly) optimized. Even though the gradient information metric (5) first proposed by Shim can be sensitive to high noise levels, it was found in this work to be an acceptable proxy for VO performance. Compared to the other metrics tested in this work, it exhibits the best linear correlation with the mean VO localization error. This conclusion contrasts some of the existing literature advocating the superiority of other metrics, but to the authors’ knowledge, no other work has previously compared metrics based on an extensive direct cross-correlation with VO performance. For instance, Kim’s [17] benchmark involves comparing the saturation rate of images defined as optimal according to the different metrics. Zhang [15] compares metrics based on the number of FAST features detected in the “best” image of different standard datasets (where the “best” image is selected as the one with the highest score and varies according to which metric is used). This approach still does not directly relate metric values to VO performance. Shin [18] uses an approach which is closest to this work by directly comparing the absolute pose error associated with images selected according to the different metrics. The authors conclude that the quality metric $M_{q}$ is a better proxy of VO performance as the best images predicted by other metrics tend to be highly noisy. However, the underlying assumption is that AE algorithms optimize a metric over the whole parameter space. Yet most AE algorithms, including this one, avoid the problem by optimizing the metric over the exposure level first. A different strategy is then used to balance gain and exposure time.

### 4.2. Prediction Models

As underlined in Section 1.2, existing AE algorithms use different models to predict the effects of future exposure parameters. Results presented in this work support using a prediction model based on the camera’s PRF rather than γ transformations due to the bias introduced by the latter. Although Zhang [15] also relies on the camera’s PRF to predict optimal changes in exposure level, the authors only evaluate the gradient of the metric at the current exposure level to inform the size of a gradient descent step. Similar to Shim’s approach, the proposed algorithm uses a set of discrete mappings based on the PRF (20) which greatly increase the range over which predictions are valid.

### 4.3. Computational Efficiency

Despite using a rough optical flow and a set of discrete simulations to drive the selection of exposure parameters, the C++ implementation of the proposed algorithm is able to run at 60 Hz on a single core of an AMD Ryzen 7 3700x (3.60 GHz) using a downsized image resolution of 360 × 270 pixels for simulations and 90 × 68 pixels for the optical flow. This real-time performance is competitive with other standard AE algorithms. Indeed, this is similar to the performance obtained by Shim [16], who reported achieving 70 Hz using an Intel Core i5-6260U (1.80 GHz) for a downsized image resolution of 320 × 240 pixels. While Tomasi [20] was able to reach a processing rate of 640 Hz using a trained CNN, the algorithm was implemented on an NVIDIA GeForce GTX 1050 Ti GPU. Reimplementing the proposed algorithm on a GPU (which are known to be 1–2 orders of magnitude faster than CPUs for image processing) would likely yield a similar processing rate.

Some AE algorithms can, however, achieve significantly lower processing times, which might prove more useful for some applications that require high frame rates with limited computational power (e.g., high-speed VO on drones). For these applications, the higher frame rate enabled by the quicker AE algorithm might offset the limitation of a suboptimal selection of the camera exposure parameters. For instance, the AE algorithm built in most modern cameras requires negligible run time as it implements a PI controller on the difference between the average pixel intensity and some reference value. Similarly, the AE control policies proposed by Kim [17] (Gaussian inference) and Shin [18] (Nelder–Mead optimization) also require minimum processing times with respect to the computation time of the actual optimization metric employed. For instance, Shin reported a computation time of <0.01 ms for a step of the Nelder–Mead method compared to the 3.23 ms required to compute the gradient-based metric of an image downsized to 800 × 600 px running on an i7-7700HQ (2.80 GHz). Both methods, however, have the downside that they require the use of query images before they can converge. These query images lead to large, oscillating changes in exposure parameters which can be detrimental for VO. Finally, while Zhang [15] does not provide details on the computational performance of the method, the algorithm does involve a few more steps, such as transforming each pixel intensity with the inverse of the camera’s PRF, computing the gradients of both the image and the transformed image, and ordering the list of the derivative of the gradient magnitudes at each pixel.

### 4.4. Saturation Feedback

To the authors’ knowledge, no other AE algorithm incorporates feedback on the difference between the predicted and the actual image metric to compensate for prediction errors due to saturated pixels. These prediction errors are especially predominant when the scene undergoes large and sudden changes in illumination. For instance, saturation feedback was shown to improve the algorithm’s speed of convergence after the lights in a room are turned on or off. However, one limitation of the method is that it adjusts the change in exposure level between the current and the next frame based on the difference between the current image metric and the one previously predicted. This delay can lead the proposed AE algorithm to sporadically overshoot the optimal exposure level, especially for larger values of the step size control parameter $f_{d}$.

## 5. Conclusions

Overall, the proposed AE algorithm was shown through experimental validation to perform at least as well as (and, on average, better than) other exposure control approaches under different challenging light conditions. One limitation of this validation is that it relies on the use of ORB-SLAM3 [27], which is known to be relatively insensitive to image noise. Future work should therefore include validation of the algorithm with VO/SLAM pipelines that are more sensitive to noise, such as direct methods like DSO [29]. Indeed, some of the design choices made in this work, including the hyperparameter *w* and the order of the exponents associated to the terms in (23), were made specifically for ORB-SLAM3 and may not be applicable to other VO methods. Another limitation of this work is that it assumes that a robot relies entirely on vision for localization. In practice, a suite of sensors (e.g., IMU, wheel encoders, LiDAR) can compensate for some of the inaccuracies of VO pipelines. Future work should therefore also explore the contribution to the proposed AE algorithm when information from other sensing modalities are also present.

## Figures and Tables

**Table 1:** AE algorithms for VO/SLAM applications.

Ref.	Year	Optimization Metric	Prediction Model	Gain/Exposure Balance Strategy	Control Policy
[12]	2009	Average intensity deviation from ref.	None	Exposure priority	PI controller
[13]	2010	Image entropy (2)	None	Preset relationship	Unspecified
[14]	2013	Average intensity deviation from ref.	Implicit	Implicit	Learned (randomly connected NN)
[15]	2017	“Soft” gradient percentile	Photometric response	Exposure priority	Gradient descent
[16]	2018	Gradient information (5)	γ transformations	Exposure priority	Nonlinear feedback minimizing $\left(\gamma^{*}-1\right)$
[17]	2018	Average entropy-weighted gradient (12)	Implicit	Fixed gain	Learned (Gaussian process)
[18]	2019	Combined gradient information, entropy, and noise metric (14)	None	Implicit	Nelder-Mead
[19]	2020	Gradient information (5)	γ transformations	Exposure priority	Nonlinear feedback minimizing $\left(\gamma^{*}-1\right)$
[20]	2021	Number of detected features and matched features across frames	Implicit	Implicit	Learned (CNN)
Ours	2021	Gradient information (5)	PRF-based transformations and saturation feedback	Minimizing motion blur and noise	Linear feedforward

## Data Availability

The data presented in this study are openly available and can be found here: (https://github.com/MIT-Bilab/vo-autoexpose accessed on 30 November 2021).

## Supplementary Materials

A supplementary video comparing the proposed algorithm with other AE methods is available online (https://youtu.be/Guvhvb-uQpE accessed on 30 November 2021).

## Abbreviations

The following abbreviations are used in this manuscript:

AE	Auto-exposure
CNN	Convolutional neural network
HDR	High dynamic range
IMU	Inertial measurement unit
PRF	Photometric response function
ROI	Region of interest
SLAM	Simultaneous localization and mapping
VO	Visual odometry
